# Impact of Static-Oriented Electric Fields on the Kinetics of Some Representative Suzuki–Miyaura and Metal-Cluster Mediated Reactions

**DOI:** 10.3390/molecules28166169

**Published:** 2023-08-21

**Authors:** Navya Arepalli, Sukanta Mondal, Debdutta Chakraborty, Pratim Kumar Chattaraj

**Affiliations:** 1 Department of Chemistry, Birla Institute of Technology, Mesra, Ranchi 835215, Jharkhand, India; 2 Department of Education, A. M. School of Educational Sciences, Assam University, Silchar 788011, Assam, India

**Keywords:** oriented electric field, kinetics, Suzuki–Miyaura reaction, metal cluster, transition state stabilization

## Abstract

In order to examine the effect of oriented (static) electric fields (OEF) on the kinetics of some representative Suzuki–Miyaura and metal-cluster mediated reactions at ambient temperatures, density functional theory-based calculations are reported herein. Results indicate that, in general, OEF can facilitate the kinetics of the concerned reactions when applied along the suitable direction (parallel or anti-parallel with respect to the reaction axis). The reverse effect happens if the direction of the OEF is flipped. OEF (when applied along the ‘right’ direction) helps to polarize the transition states in the desired direction, thereby facilitating favorable bonding interactions. Given the growing need for finding appropriate catalysts among the scientific community, OEF can prove to be a vital route for the same.

## 1. Introduction

One of the main goals of chemistry is to achieve desired reactivity changes within molecules by suitably tuning the external physicochemical conditions. To this end, suitable catalysts are usually used in order to achieve kinetic acceleration for a given chemical reaction. Of late, several other avenues for catalyzing chemical reactions are being actively explored by several research groups. Prominent routes among them are the effects of an external electric field [1,2,3,4,5,6,7,8,9,10] and the effects of confinement [11,12,13,14,15,16], etc. Chemical reactions can often lead to multiple product formation. The reasons for product branching effects could be due to the thermodynamic/kinetic or dynamic (i.e., time-dependent) factors [17,18,19,20,21,22,23]. Controlling the product branching effects is an important objective in research so that the reaction can be driven toward the desired product formation route. To this end, the impact of time-dependent oscillating electric/laser fields on reaction dynamics has been explored in the past from both theoretical (using model potentials as well as using small molecules) and experimental points of view [24,25,26,27,28,29]. Of late, the impact of oriented (static) electric fields (OEFs) has also been studied within a time-independent framework by several researchers to gauge the effect of the same in making chemical reactions more facile from a kinetic perspective [1,2,3,4,5,6,7,8,9,10]. For example, Meir et al. [1] have computationally demonstrated the ability of OEF to catalyze some representative Diels–Alder reactions. Experimental evidence [6] for the catalysis of some Diels–Alder reactions by an OEF was provided by Coote et al. Timerghazin et al. have computationally demonstrated that the site preference for nucleophilic attacks on S-nitrosothiols could be controlled by the application of OEFs [2]. Shaik et al. have shown that the selectivity of two competing reaction channels, i.e., C=C epoxidation and C–H hydroxylation within cytochrome P450, could be controlled by the application of an OEF [3]. Bhattacharyya et al. computationally showed that the Huisgen reaction between alkyl azide and cyclooctyne (biflurocyclooctyne) could be kinetically accelerated by the application of a suitable OEF [5].

During the course of chemical reactions, maximum changes in the geometrical features of the reactants happen along the main reaction coordinate. Therefore, through the application of an external perturbation, such as an OEF, one could, in principle, force the reaction to proceed in the desired direction, as the OEF could facilitate the suitable geometrical re-arrangements required by the reactants. However, given the fact that an OEF is a vector quantity, a reverse effect might happen if the direction of the OEF is altered. Nonetheless, only geometrical considerations might not be the only factor that impacts the fate of a given chemical reaction’s kinetics in the presence of an OEF. There could be subtle quantum electronic factors that might play significant roles in these processes, as the OEF also has the ability to affect the molecular orbitals of the reactants. Thus, detailed quantum chemical analyses might be needed to understand the impact of OEFs on a given chemical reaction. 

In this work, we report an exploratory study on the impact of OEFs on some representative Suzuki–Miyaura cross-coupling [30,31,32,33,34] and metal cluster-mediated reactions [35] with the help of density functional theory (DFT) based calculations. It is a well-known fact in the literature that the Suzuki–Miyaura reaction constitutes one of the most prominent routes for achieving cross-coupling in organic chemistry [30,31,32,33,34]. The concerned reaction mechanism usually involves two competing pathways: oxidative addition and nucleophilic displacement [34]. It has been suggested in the literature that these cross-coupling reactions can also proceed through radical pathways [34]. However, in simple model systems (which have been considered in this work), oxidative addition and nucleophilic displacement seem to play significant roles [33]. Thus, we consider these two pathways in our present analysis. To this end, we have considered the following four reactions (in Tetrahydrofuran (THF) solvent): (1) PhCl + Pd(PMe_3_), (2) PhCl + Pd(PMe_3_)_2_, (3) PhBr + Pd(PMe_3_), and (4) PhBr + Pd(PMe_3_)_2_. On the other hand, we have also considered (in the gas phase) the (5) C-F bond activation in CH_3_F mediated by the Al_12_Be metal cluster [35] as a representative example. In all the aforementioned chemical reactions, the impact of OEFs in affecting the free energy of activation has been examined by employing the OEF in parallel and anti-parallel orientation with respect to the reaction coordinate. In particular, our emphasis in this study is to understand how OEFs affect the energetics of the transition states in the concerned reactions. In what follows, we describe the results and discussion, followed by the computational details adopted in our work. 

## 2. Results and Discussion

### 2.1. Effect of OEFs on the Free Energy of Activation $\left(∆G^{‡}\right)$ (Suzuki–Miyaura Coupling Reactions)

We begin our discussion by considering the results obtained for the Suzuki–Miyaura coupling reactions (reactions 1 to 4). All these reactions involve different transition states due to the possibility of two mechanistic pathways, namely, concerted and displacement. In all these reactions (1 to 4), the effect of an OEF on the free energy of activation has been studied with respect to all the transition states that were located for a particular reaction.

**Figure 1:**
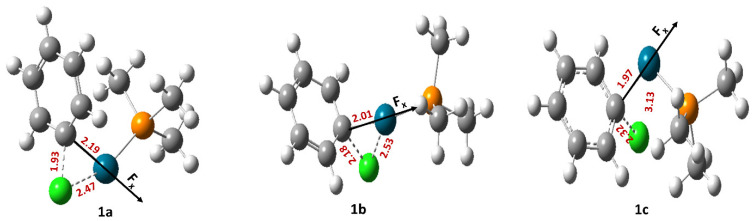
The optimized geometries of the transition states involved in reaction 1. Here, the grey color denotes C, the green color denotes Cl, the white color denotes H, the orange color denotes P, and the blue color denotes Pd atoms, respectively.

For reaction 1, three different transition states were located, namely 1a, 1b, and 1c (Figure 1). 

Among these three, 1a and 1b represent the concerted mechanism, whereas 1c represents the displacement mechanism [33]. When the reaction proceeds through the transition state 1a (Table 1), the free energy of activation $\left(∆G^{‡}\right)$ always increases irrespective of the direction of the OEF but for one case.

In the cases of transition states 1b and 1c, however, the free energy of activation decreases when the OEF is applied parallel to the reaction coordinate. The magnitude of the decrease in free energy of activation increases with an increase in the strength of the OEF applied. The reverse effect happens when the OEF is applied in parallel orientation with respect to the reaction coordinate.

**Figure 2:**
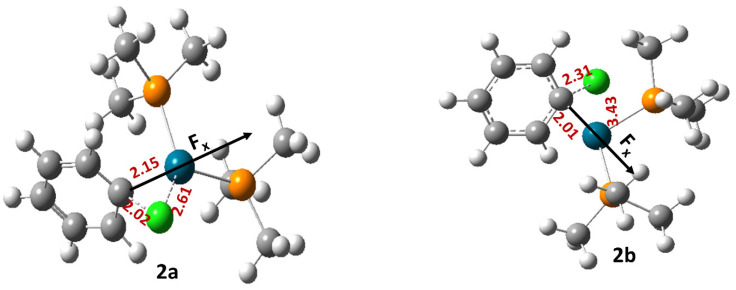
The optimized geometries of the transition states involved in reaction 2. Here, the grey color denotes C, the green color denotes Cl, the white color denotes H, the orange color denotes P, and the blue color denotes Pd atoms, respectively.

For reaction 2, two different transition states were located, namely 2a and 2b (Figure 2). 

Among these two, 2a represents the concerted mechanism, whereas 2b represents the displacement mechanism [33]. An OEF applied in an anti-parallel orientation with respect to the reaction coordinate reduces the free energy of activation $\left(∆G^{‡}\right)$ of reaction 2 when it proceeds through both the transition states 2a and 2b (Table 2). Flipping the orientation of an OEF (parallel in case of both 2a and 2b) increases the free energy of activation $\left(∆G^{‡}\right)$ with respect to the zero field conditions. 

**Figure 3:**
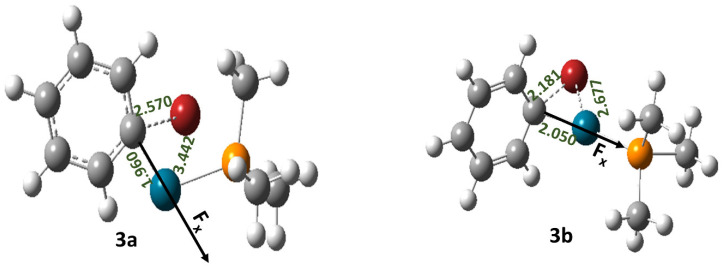
The optimized geometries of the transition states involved in reaction 3. Here, the grey color denotes C, the red color denotes Br, the white color denotes H, the orange color denotes P, and the blue color denotes Pd atoms, respectively.

In the case of reaction 3, two different transition states were located, namely 3a and 3b (Figure 3). 

Herein, 3a represents the displacement mechanism, whereas 3b represents the concerted mechanism [34]. An OEF applied in anti-parallel orientation with respect to the reaction coordinate reduces the free energy of activation $\left(∆G^{‡}\right)$ of reaction 3 when it proceeds through both the transition states 3a and 3b, whereas the reverse effect happens when the direction of the OEF is altered (Table 3).

**Figure 4:**
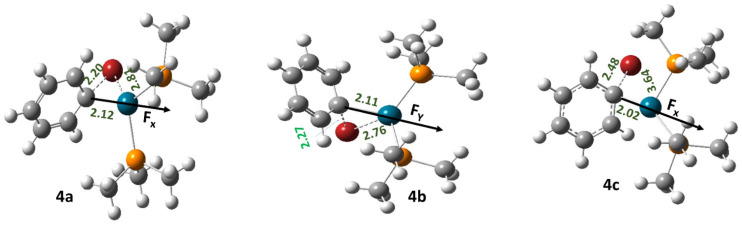
The optimized geometries of the transition states involved in reaction 4. Here, the grey color denotes C, the red color denotes Br, the white color denotes H, the orange color denotes P, and the blue color denotes Pd atoms, respectively.

For reaction 4, three different transition states were located, namely 4a, 4b, and 4c (Figure 4). 

Herein, 4a and 4b represent the concerted mechanism, whereas 4c represents the displacement mechanism [34]. Generally, an OEF applied in parallel orientation with respect to the reaction coordinate reduces the free energy of activation $\left(∆G^{‡}\right)$ with respect to the zero field conditions of reaction 4 when it proceeds through the transition states 4a and 4b. In case 4c, however, the free energy of activation $\left(∆G^{‡}\right)$ increases irrespective of the direction of OEF (Table 4).

In all the aforementioned cases, the OEF polarizes the concerned transition states quite significantly. However, the direction in which the polarization happens in these transition states is dependent on the orientation of the OEF. To this end, we consider the component of the dipole moment along the reaction coordinate ($\mu_{x}/\mu_{y}$). It can be seen from Table 1, Table 2, Table 3 and Table 4 that the magnitude of $\mu_{x}/\mu_{y}$ generally changes significantly in the presence of increasing strength of the OEF (irrespective of the direction of the OEF) as compared to the unperturbed transition states. The crucial factor, however, is being played by the direction of $\mu_{x}$. If the OEF polarizes the transition states in the direction in which the new bond is about to be formed, the reactions generally become kinetically more favorable. The reverse effect happens if the direction of the OEF is flipped. 

### 2.2. Can OEFs Change the Reaction Mechanism?

**Figure 5:**
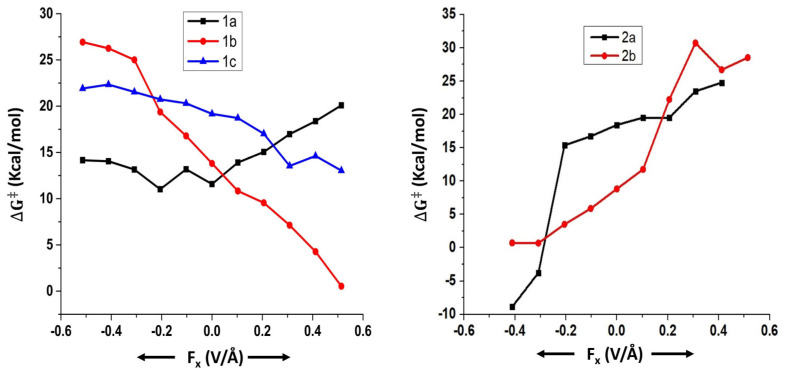
Variation of free energy of activation $\left(∆G^{‡}\right)$ as a function of OEFs for different pathways in reactions 1 and 2.

**Figure 6:**
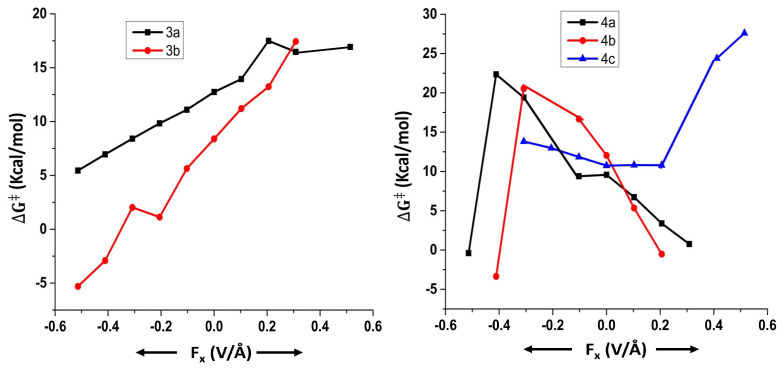
Variation of free energy of activation $\left(∆G^{‡}\right)$ as a function of OEF for different pathways in reactions 3 and 4.

We note that the objective of the application of OEFs in a given chemical reaction is not only to check whether the concerned reaction becomes more favorable from the kinetic perspective, but the OEF could also be utilized to control the product selectivity. In order to explore this aspect, we discuss the following results (Figure 5 and Figure 6). 

For reaction 1, in the absence of an OEF, the concerted mechanism is favored over the displacement mechanism. On application of an OEF, this trend does not change irrespective of the direction of the OEF.

For reaction 2, in the absence of an OEF, the displacement mechanism is favored over the concerted mechanism. At high field strengths at both parallel and anti-parallel directions, there is a mechanistic crossover, and the concerted mechanism is favored over the displacement mechanism.

For reaction 3, in the absence of an OEF, the concerted mechanism is favored over the displacement mechanism. However, the mechanistic crossover can happen at high field strengths of the OEF in parallel orientation.

For reaction 4, in the absence of an OEF, the displacement mechanism is favored over the concerted mechanism. Nonetheless, the mechanistic crossover could happen at high field strengths of an OEF applied along the anti-parallel orientation. However, in case 4, several calculations did not converge, and thus, we mention these results with caution. 

Based on the aforementioned facts, we can infer that OEF can indeed be used in certain cases to drive the reaction in the desired direction. 

### 2.3. Effect of OEFs on Free Energy of Activation $\left(∆G^{‡}\right)$ (Metal Cluster-Mediated Reactions)

**Figure 7:**
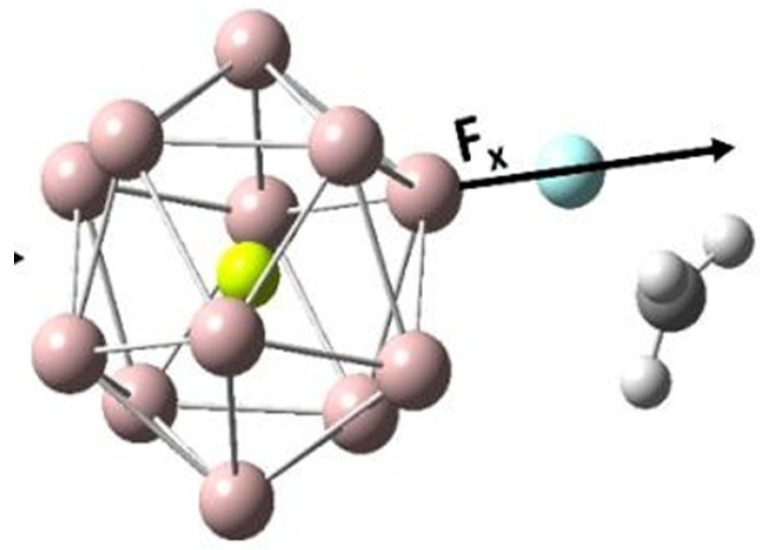
The optimized geometry of the transition state involved in reaction 5. Here, the yellow color denotes Be, the pink color denotes Al, the white color denotes H, the blue color denotes F atoms, and the grey color denotes C, respectively.

We now consider reaction 5, i.e., (5) C–F bond activation in CH_3_F mediated by the Al_12_Be metal cluster (Table 5, Figure 7).

In this reaction, the free energy of activation $\left(∆G^{‡}\right)$ generally reduces when the OEF is applied along the parallel direction. The reverse effect happens if the OEF is flipped in both the cases. Noting the direction of the component of the dipole moment along the reaction coordinate ($\mu_{x}$), we can infer that the OEF helps to polarize the transition states in the direction of the bond formation. 

Based on the aforementioned discussions, it becomes clear that the OEF (when applied along the ‘desired’ direction) can quite effectively facilitate the concerned reactions. Obviously, the transition states become stabilized/de-stabilized from energetic points of view as a function of the direction of the OEF. To shed some light on the impact of OEF on the bonding interactions present within the concerned transition states, we now discuss the results obtained from EDA.

### 2.4. Results Obtained from EDA

The total interaction energy within a given system could be decomposed into the orbital interaction (E_orb_), the exchange interaction (E_ex_), and the electrostatic interaction (E_els_). The summation of exchange and the electrostatic interaction could be expressed as the steric interaction. We have performed the energy decomposition analysis for all the transition states reported herein (for all the transition states as obtained in the presence and in the absence of external electric fields). We have presented the corresponding results in the supporting information (Figures S1–S7). It becomes quite evident that the primary driving force for the stabilization of the concerned transition states is the favorable orbital interaction term. Orbital interaction term reflects the polarization and bonding interactions present within the system. OEF facilitates the geometrical arrangement of the concerned transition states so that the bonding interaction between the intervening fragments becomes more favorable (in the cases where an OEF kinetically facilitates the reactions). However, the reverse effect happens when the direction of the OEF is altered, and the bonding interaction becomes less favorable as compared to the cases where no external perturbation is present. 

In summary, OEFs facilitate the polarization and bonding interactions along the main reaction axis, thereby imparting a stabilizing influence on the transition states.

## 3. Materials and Methods

The molecular modeling (reported in this work) has been carried out using Gauss View 6.0 software [36]. All the transition states and reactants corresponding to reactions 1–4 in the presence as well as in the absence of a static-oriented electric field (OEF) have been optimized using the MN15L [37] functional along with 6-311G(d,p) basis set for C, H, Cl, Br, and P. For Pd, the LANL2DZ basis set has been used by taking into consideration the concerned effective core potential (ECP). Here, for reactions 1 to 4, we have chosen the MN15L functional as it was proven to perform well for reactions 1 to 4 by previous studies [33]. For reactions 1 to 4, the solvent effect of tetrahydrofuran (THF) was incorporated by using the Polarizable Continuum solvation Model (PCM) [38]. For reaction 5, the geometry optimization has been performed at the wb97xd [39] level of theory (as was performed in our previous studies on this system) along with the basis set 6-311++G(d,p). No constraints have been imposed while performing the geometry optimizations. To examine the nature of all the stationary points on the concerned potential energy surface (PES), harmonic vibrational frequencies have been computed. The reactants contain only real-valued vibrational frequencies, whereas the transition states contain only one imaginary vibrational frequency. The intrinsic reaction coordinate (IRC) method has been utilized to ascertain that the concerned transition states are connected to the respective reactant and product structures. All the aforementioned calculations have been performed with the help of the Gaussian 16 code [40]. 

To study the effect of a static external electric field on the free energy of activation of all the reactions that are considered here, a static electric field was applied along the reaction axis (in both parallel as well as anti-parallel directions) using the keyword ‘Field’ (in conjunction with the NOSYMM keyword so as to prevent the re-orientation of the concerned molecules), as implemented in the Gaussian 16 code [40]. In order to compute the free energy of activation, the preceding local minima (to the transition state) have been considered as the reference reactant state [1] (both in the presence and absence of an OEF at 298 K). To this end, the last point on the reactant’s side from the IRC scan was optimized (in conjunction with frequency calculation). We have applied the OEF parallelly (and anti-parallelly) with respect to the reaction axis. The principle physical logic for the choice of our computational protocol is the underlying assumption of the validity of the minimum energy path (MEP). Herein, we assume that all the concerned reactions follow MEP. By visualizing (via Gauss View 6.0 software) the IRC scan, as well as the transition state and reactant complex geometries, the suitable reaction axis was defined, and the OEF was applied with respect to this axis. Eyring’s equation has been used to calculate (qualitative) rate constants. To understand the principle electronic factors that stabilize/de-stabilize the transition states, energy decomposition analysis (EDA) has been performed using the Multiwfn software [41] in conjunction with the Gaussian 16 code (at the same level of theory which has been mentioned above). 

## 4. Conclusions

In this work, we have tried to understand the effect of static OEF on the kinetics of some representative Suzuki–Miyaura and some metal cluster-mediated reactions with the aid of density functional theory based calculations. Results indicate that the concerned reactions could be kinetically facilitated when the OEF is applied in a suitable direction but for two cases. The reactions, however, become hindered when the direction of the OEF is flipped. The crucial factor that has emerged from our analyses is the ability of the OEF to polarize the transition states in the bond-formation direction. OEFs also facilitate the bonding interactions within the concerned transition states, as evidenced by the energy decomposition analysis. Due to these factors, the transition states become energetically stabilized (in the presence of an OEF) as compared to the corresponding situation in the absence of any external perturbation. We have also demonstrated that, in certain cases (when multiple reaction pathways are available), OEFs can drive the reaction toward a particular pathway. Therefore, product selectivity, at least in principle, could be controlled via the application of an OEF. As the considered strengths of the OEF are well within the reach of several experimental setups [4,5], it might be worthwhile to check the validity of the work presented here from an experimental point of view.

## Figures and Tables

**Table 1:** The changes in the free energy of activation and the x-component of the dipole moment for the transition state involved in reaction 1 as a function of the OEF.

Field Strength (Fx) (V/Å)	Dipole Moment (*μ_x_*) (D) (1a)	Free Energy of Activation (∆*G*^‡^) (kcal/mol) (1a)	Rate Constant (k, s^−1^) (1a)	Dipole Moment (*μ_x_*) (D) (1b)	Free Energy of Activation (∆*G*^‡^) (kcal/mol) (1b)	Rate Constant (k, s^−1^) (1b)	Dipole Moment (*μ_x_*) (D) (1c)	Free Energy of Activation (∆*G*^‡^) (kcal/mol) (1c)	Rate Constant (k, s^−1^) (1c)
−0.5140	9.90	14.2	2.6 × 10^2^	8.02	26.9	1.1 × 10^−7^	5.91	21.9	5.4 × 10^−4^
−0.4112	8.03	14.0	3.1 × 10^2^	5.16	26.3	3.5 × 10^−7^	3.62	22.3	2.6 × 10^−4^
−0.3084	6.39	13.2	1.4 × 10^3^	2.98	25.0	2.9 × 10^−6^	1.43	21.5	1.0 × 10^−3^
−0.2056	5.12	11.0	5.2 × 10^4^	0.71	19.4	3.9 × 10^−2^	−0.66	20.7	3.9 × 10^−3^
−0.1028	4.05	13.2	1.4 × 10^3^	−1.34	16.8	3.1	−2.07	20.3	8.1 × 10^−3^
0	3.20	11.6	2.0 × 10^4^	−3.55	13.8	4.7 × 10^2^	−4.72	19.2	5.5 × 10^−2^
0.1028	2.25	13.9	3.9 × 10^2^	−5.10	10.8	7.1 × 10^4^	−6.75	18.7	1.2 × 10^−1^
0.2056	1.13	15.1	5.7 × 10^1^	−6.90	9.6	6.2 × 10^5^	−8.77	17.0	2.1
0.3084	−0.08	17.0	2.2	−8.70	7.1	3.7 × 10^7^	−10.86	13.5	7.3 × 10^2^
0.4112	−1.36	18.4	2.1 × 10^−1^	−10.63	4.3	4.6 × 10^9^	−12.96	14.6	1.2 × 10^2^
0.5140	−2.70	20.1	1.2 × 10^−2^	−12.77	0.5	2.5 × 10^12^	−15.03	13.0	1.7 × 10^3^

**Table 2:** The changes in the free energy of activation and the x-component of the dipole moment for the transition state involved in reaction 2 as a function of the OEF.

Field Strength (Fx) (V/Å)	Dipole Moment (*μ_x_*) (D) (2a)	Free Energy of Activation (∆*G*^‡^) (kcal/mol) (2a)	Rate Constant (k, s^−1^) (2a)	Dipole Moment (*μ_x_*) (D) (2b)	Free Energy of Activation (∆*G*^‡^) (kcal/mol) (2b)	Rate Constant (k, s^−1^) (2b)
−0.5140	-	***	-	-	***	-
−0.4112	16.57	−8.9	2.0 × 10^19^	18.12	0.7	1.8 × 10^12^
−0.3084	16.22	−3.8	3.5 × 10^15^	16.92	0.7	1.9 × 10^12^
−0.2056	8.53	15.4	3.3 × 10^1^	15.30	3.5	1.6 × 10^10^
−0.1028	6.00	16.7	3.3	13.28	5.9	3.0 × 10^8^
0	3.50	18.4	1.9 × 10^−1^	11.03	8.8	2.1 × 10^6^
0.1028	1.05	19.5	3.0 × 10^−2^	8.38	11.8	1.5 × 10^4^
0.2056	−2.33	19.5	3.1 × 10^−2^	5.61	22.3	3.0 × 10^−4^
0.3084	−4.99	23.5	3.8 × 10^−5^	2.30	30.7	1.8 × 10^−10^
0.4112	−7.51	24.8	4.2 × 10^−6^	−7.56	26.7	1.6 × 10^−7^
0.5140	-	***	-	−10.20	28.5	7.5 × 10^−9^

*** Converged results could not be obtained.

**Table 3:** The changes in the free energy of activation and the x-component of the dipole moment for the transition state involved in reaction 3 as a function of the OEF.

Field Strength (Fx) (V/Å)	Dipole Moment (*μ_x_*) (D) (3a)	Free Energy of Activation (∆*G*^‡^) (kcal/mol) (3a)	Rate Constant (k, s^−1^) (3a)	Dipole Moment (*μ_x_*) (D) (3b)	Free Energy of Activation (∆*G*^‡^) (kcal/mol) (3b)	Rate Constant (k, s^−1^) (3b)
−0.5140	15.04	5.4	6.3 × 10^8^	11.98	−5.3	4.8 × 10^16^
−0.4112	12.90	6.9	5.0 × 10^7^	10.22	−2.9	8.4 × 10^14^
−0.3084	10.78	8.4	4.2 × 10^6^	8.47	2.0	2.0 × 10^11^
−0.2056	8.64	9.8	3.9 × 10^5^	6.84	1.1	9.2 × 10^11^
−0.1028	6.50	11.1	4.6 × 10^4^	5.20	5.6	4.6 × 10^8^
0	4.37	12.7	2.8 × 10^3^	2.85	8.4	4.4 × 10^6^
0.1028	2.21	13.9	3.7 × 10^2^	0.89	11.2	3.8 × 10^4^
0.2056	0.07	17.5	9.3 × 10^−1^	−1.27	13.2	1.2 × 10^3^
0.3084	−2.10	16.5	5.3	−3.52	17.4	1.0
0.4112	-	#	#	−5.72	#	-
0.5140	−6.65	16.9	2.4	-	#	-

# The reactant geometry could not be converged after several attempts.

**Table 4:** The changes in the free energy of activation and the x/y-component of the dipole moment for the transition state involved in reaction 4 as a function of OEF.

Field Strength (Fx) (V/Å)	Dipole Moment (*μ_x_*) (D) (4a)	Free Energy of Activation (∆*G*^‡^) (kcal/mol) (4a)	Rate Constant (k, s^−1^) (4a)	Dipole Moment (*μ_y_*) (D) (4b)	Free Energy of Activation (∆*G*^‡^) (kcal/mol) (4b)	Rate Constant (k, s^−1^) (4b)	Dipole Moment (*μ_x_*) (D) (4c)	Free Energy of Activation (∆*G*^‡^) (kcal/mol) (4c)	Rate Constant (k, s^−1^) (4c)
−0.5140	12.36	−0.4	1.2 × 10^13^	15.57	***	-	***	***	-
−0.4112	6.23	22.4	2.5 × 10^−4^	13.42	−3.3	1.8 × 10^15^	***	***	-
−0.3084	4.52	19.4	3.7 × 10^−2^	11.11	20.5	5.4 × 10^−3^	3.62	13.8	4.6 × 10^2^
−0.2056	-	***	-	-	***	-	−5.49	13.0	1.9 × 10^3^
−0.1028	−0.28	9.4	8.1 × 10^5^	6.31	16.7	3.7	−9.18	11.8	1.3 × 10^4^
0	−10.72	9.6	6.0 × 10^5^	3.88	12.0	9.2 × 10^3^	−11.80	10.7	8.2 × 10^4^
0.1028	−15.53	6.7	7.2 × 10^7^	1.58	5.3	7.5 × 10^8^	−13.93	10.8	7.3 × 10^4^
0.2056	−15.93	3.4	2.0 × 10^10^	−0.73	−0.5	1.5 × 10^13^	−15.33	10.8	7.7 × 10^4^
0.3084	−15.50	0.8	1.7 × 10^12^	−3.11	***	-	-	***	-
0.4112	***	-	-	−5.56	***	-	-5.5	24.4	7.5 × 10^−6^
0.5140	***	-	-	−8.06	***	-	-8.05	27.6	3.5 × 10^−6^

*** Converged results could not be obtained.

**Table 5:** The changes in the free energy of activation and the x-component of the dipole moment for the transition state involved in reaction 5 as a function of an OEF.

Field Strength (Fx) (V/Å)	Dipole Moment (*μ_x_*) (D)	Free Energy of Activation (∆*G*^‡^) (kcal/mol)	Rate Constant (k, s^−1^)
−0.5140	10.42	43.8	5.2 × 10^−20^
−0.4112	7.68	38.9	1.8 × 10^−16^
−0.3084	4.97	34.5	3.5 × 10^−13^
−0.2056	2.25	29.7	9.7 × 10^−10^
−0.1028	−0.48	25.4	1.4 × 10^−6^
0	−3.26	20.6	4.7 × 10^−3^
0.1028	−6.06	16.2	8.6
0.2056	−8.90	11.7	1.6 × 10^4^
0.3084	−11.20	8.8	2.2 × 10^6^
0.4112	−14.81	3.8	1.0 × 10^10^
0.5140	−17.97	−0.9	3.0 × 10^13^

## Data Availability

All the scientific data reported in this manuscript are available from the corresponding authors upon reasonable request.

## Supplementary Materials

The following supporting information can be downloaded at: https://www.mdpi.com/article/10.3390/molecules28166169/s1, Figure S1: Results of EDA for reaction 1a. Figure S2: Results of EDA for reaction 1b. Figure S3: Results of EDA for reaction 1c. Figure S4: Results of EDA for reaction 2a. Figure S5: Results of EDA for reaction 2b. Figure S6: Results of EDA for reaction 3a. Figure S7: Results of EDA for reaction 3b. Figure S8: Results of EDA for reaction 4a. Figure S9: Results of EDA for reaction 4b. Figure S10: Results of EDA for reaction 4c. Figure S11: Results of EDA for reaction 5.
