# Supplementary material for: Impact of Static-Oriented Electric Fields on the Kinetics of Some Representative Suzuki–Miyaura and Metal-Cluster Mediated Reactions
Source: Molecules. 2023 Aug 21;28(16):6169. doi: 10.3390/molecules28166169 (PMC10459314; doi:10.3390/molecules28166169)
Supplement: Supplementary file 1 [file molecules-28-06169-s001.zip › molecules-2465849-supplementary.pdf]

Impact of Static-Oriented Electric Fields on the Kinetics of Some Representative Suzuki–Miyaura and Metal-Cluster Mediated Reactions

Navya Arepalli [1,†], Sukanta Mondal [2,†], Debdutta Chakraborty [1,*] and Pratim Kumar Chattaraj [1,*]

[1] Department of Chemistry, Birla Institute of Technology, Mesra, Ranchi 835215, Jharkhand, India
[2] Department of Education, A. M. School of Educational Sciences, Assam University, Silchar 788011, Assam, India
[*] Correspondence: debdutta.chakraborty@bitmesra.ac.in (D.C.); pkc@chem.iitkgp.ac.in (P.K.C.)
[†] These authors contributed equally to this work.

Supporting Information

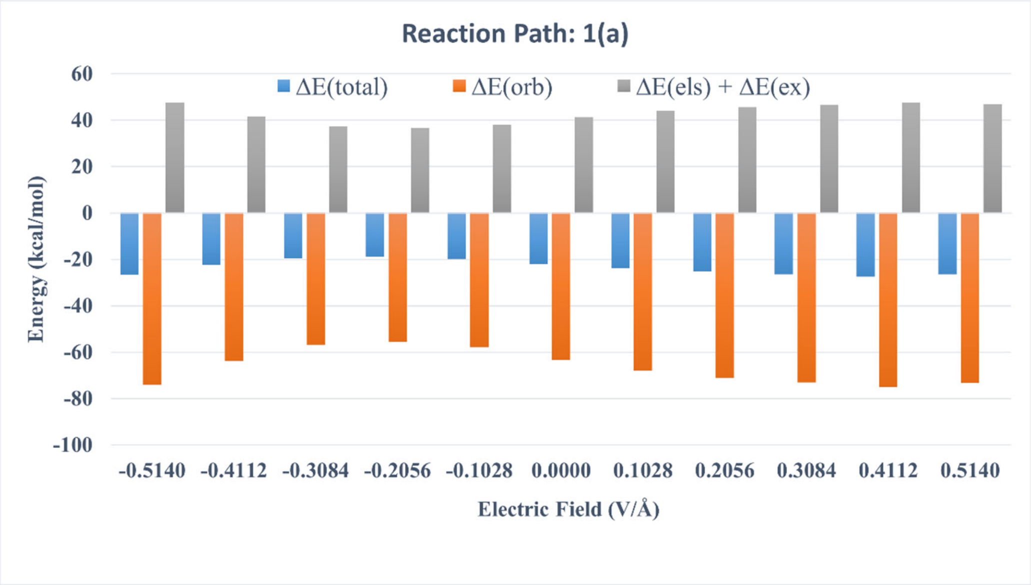

Figure S1: Results of EDA for reaction 1a.

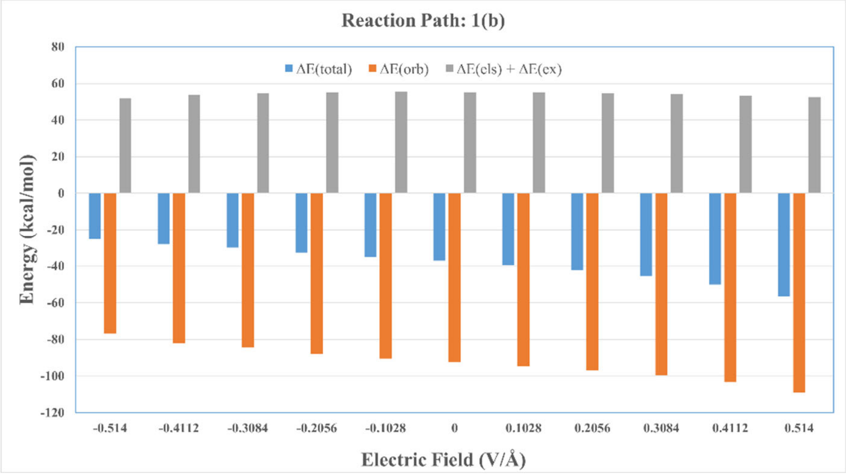

Figure S2: Results of EDA for reaction 1b.

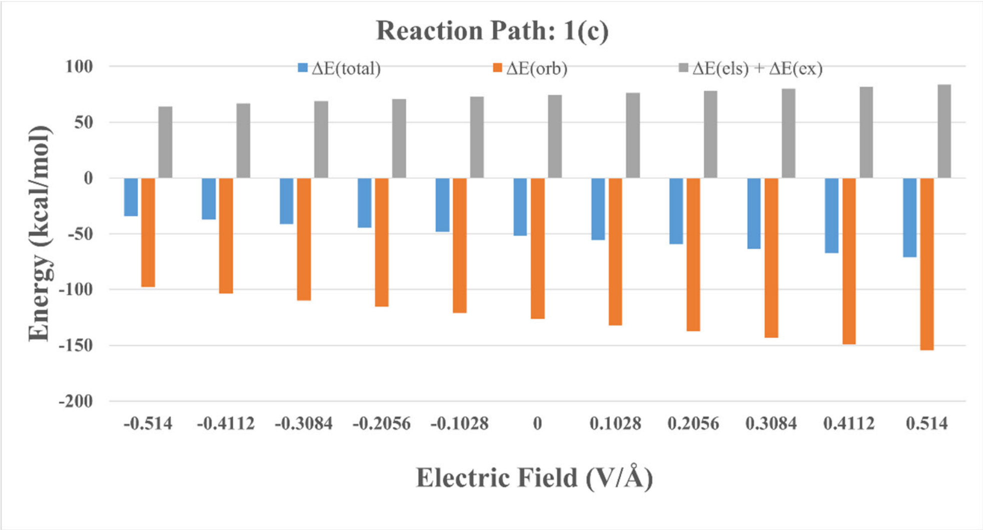

Figure S3: Results of EDA for reaction 1c.

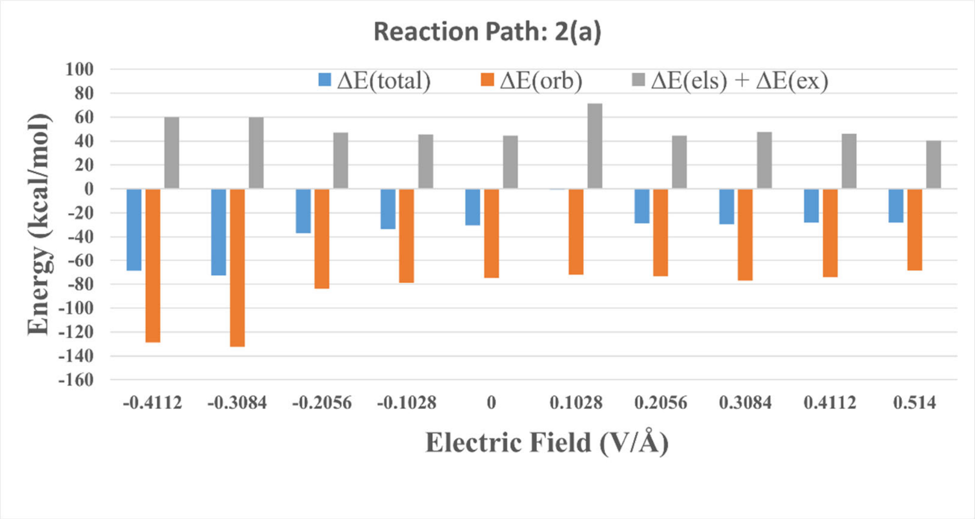

Figure S4: Results of EDA for reaction 2a.

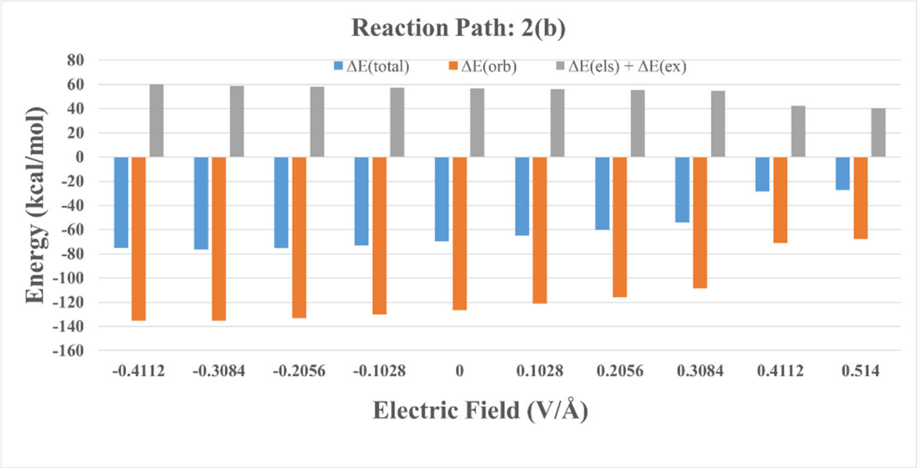

Figure S5: Results of EDA for reaction 2b.

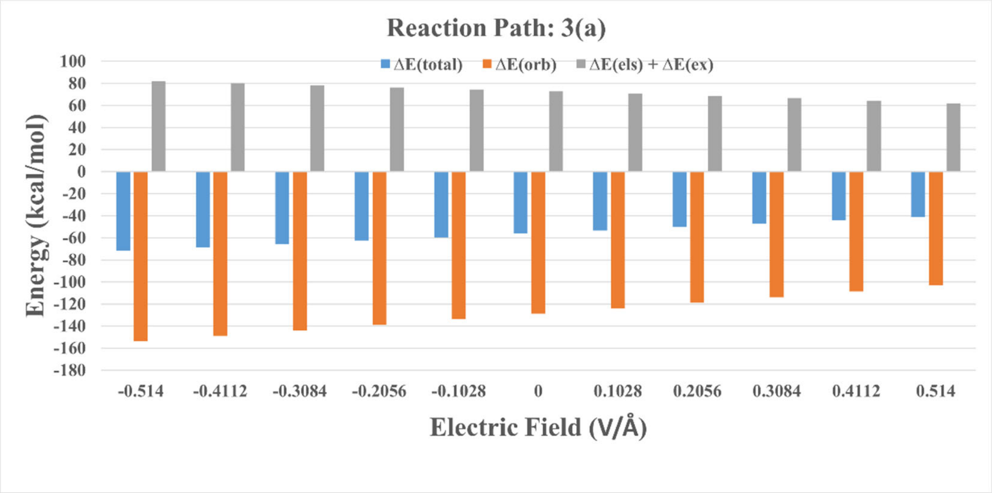

Figure S6: Results of EDA for reaction 3a**.**

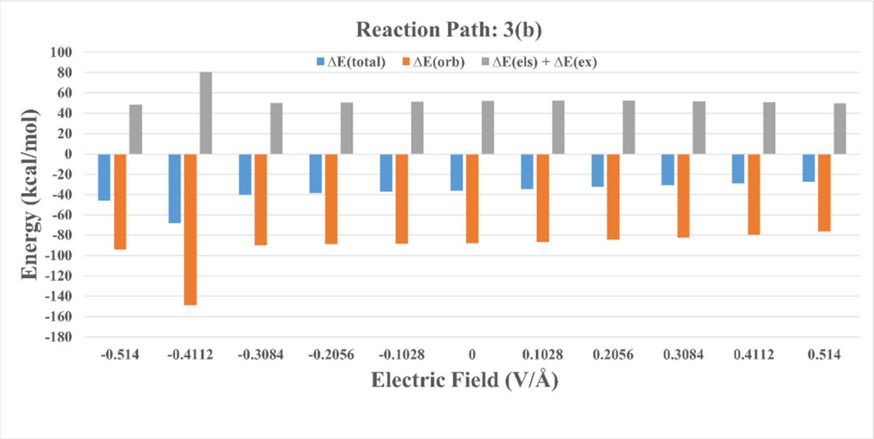

Figure S7: Results of EDA for reaction 3b.

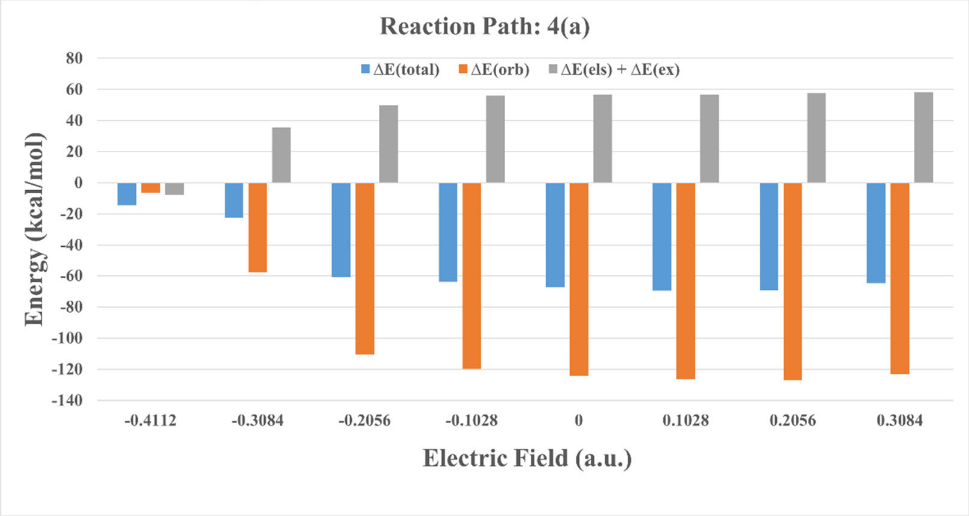

Figure S8: Results of EDA for reaction 4a.

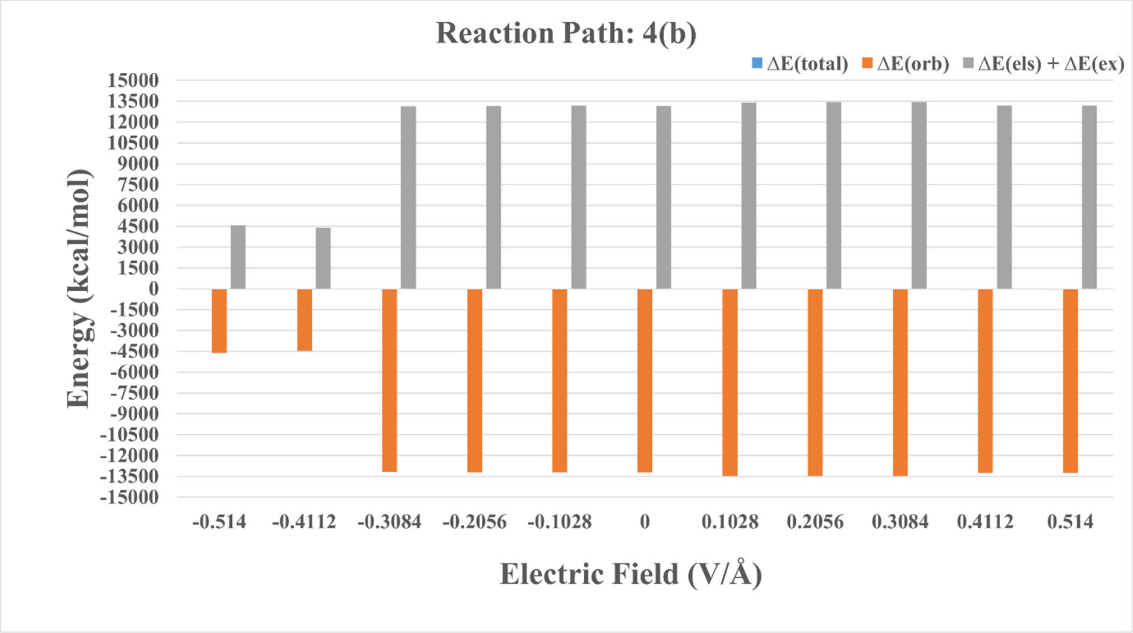

Figure S9: Results of EDA for reaction 4b.

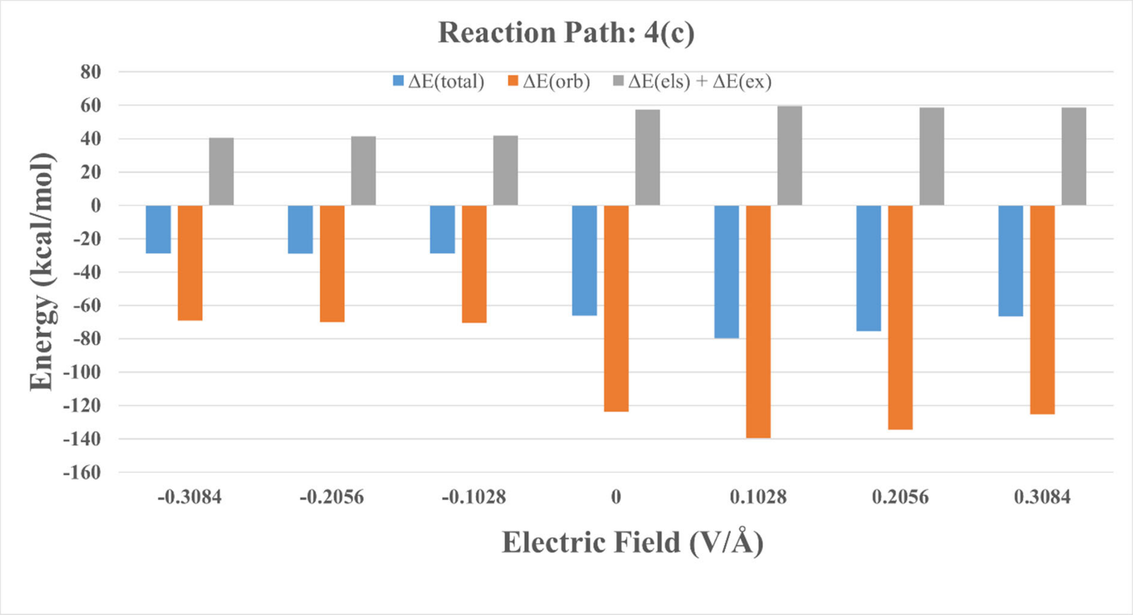

Figure S10: Results of EDA for reaction 4c.

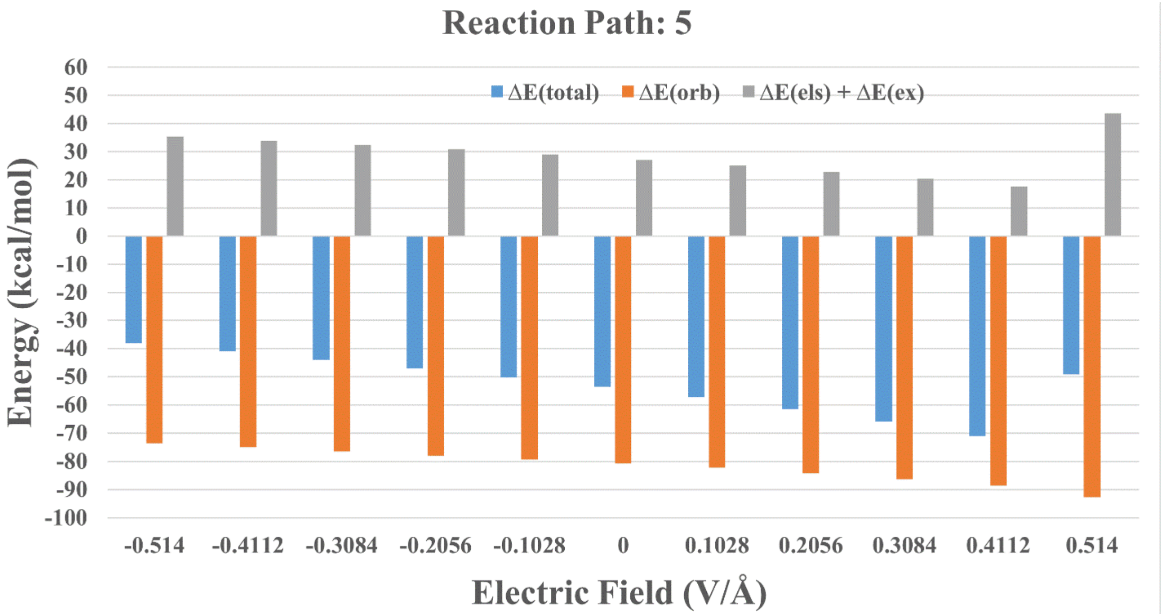

Figure S11: Results of EDA for reaction 5.
